# TANK-Binding Kinase 1 Mutation as a Rare Cause of Frontotemporal Dementia in a Mexican Patient: The First Case Report in a Tertiary Referral Hospital in Mexico

**DOI:** 10.7759/cureus.43954

**Published:** 2023-08-23

**Authors:** Humberto Estrada-Rodriguez, Daniel A Meza-Martinez, Marco Antonio Muñuzuri-Camacho, David Garcia-Romero, Isael Reyes-Melo

**Affiliations:** 1 Neurology and Psychiatry Department, National Institute of Medical Sciences and Nutrition, Mexico City, MEX; 2 Cirugía General, Instituto Mexicano del Seguro Social, Hospital General de Zona No. 33, Monterrey, MEX; 3 Neurocirugía, Instituto Nacional de Neurología y Neurocirugía Manuel Velasco Suárez, Ciudad de Mexico, MEX; 4 Christus Muguerza Hospital, Autonomous University of Nuevo Leon, Monterrey, MEX

**Keywords:** early-onset dementia, motor neuron disease, progressive supranuclear palsy (psp), gene, neurocognitive syndrome, parkinsonian disorder, frontotemporal dementia, tbk1

## Abstract

Frontotemporal dementia (FTD) is a heterogeneous condition characterized by changes in behavior, personality, and language resulting from degeneration of the frontal and/or temporal lobes. A wide spectrum of clinical syndromes and an overlap with different motor disorders make this entity challenging for clinicians, both in achieving a correct diagnosis and providing proper treatment. Despite the majority of cases being sporadic, FTD has a hereditary component, and more than 10 disease-causing genes have been identified. We present the case of a Mexican patient with a positive family history of neurocognitive disorders who developed early-onset behavioral symptoms, cognitive alterations, and motor disturbances. After a comprehensive study and multiple assessments by various medical services, a molecular diagnosis was achieved by documenting a loss-of-function mutation in the TANK-binding kinase 1 (TBK1) gene, an extremely rare cause of FTD. Genetic diagnosis is crucial in these situations, as this mutation has been associated with rapid disease progression and the potential development of motor syndromes during its course.

Our case underscores the challenges involved in reaching an accurate diagnosis, highlighting the importance of molecular testing. A thorough family history, past medical records, and a detailed description of symptom onset and progression are imperative, as they can significantly influence both treatment approaches and prognosis. Diagnostic errors, combined with their subsequent inappropriate treatment, can further deteriorate patients' quality of life.

## Introduction

FTD is a heterogeneous disorder, both clinically and neuropathologically. It is characterized by disruptions in behavior, personality, and language coupled with localized degeneration of the frontal and/or temporal lobes. FTD is an overarching term that encompasses three main clinical syndromes: behavioral variant FTD (bvFTD), semantic variant primary progressive aphasia (svPPA), and nonfluent variant primary progressive aphasia (nfvPPA) [1]. Each clinical syndrome may correspond to distinct neuropathological changes. This situation poses a notable challenge in creating and evaluating treatments targeting distinct protein dysfunctions within the framework of neurodegeneration [2].

FTD is a common cause of early-onset dementia, with a frequency similar to Alzheimer's disease (AD) in individuals under the age of 65. It typically has an average age of onset of 58 years, although cases have been reported with onsets between 30 and 80 years [3,4]. Moreover, recent research reveals a substantial overlap in neuropathology and genetics between FTD and motor neuron diseases (MND) [5].

This condition has a strong hereditary component, with recent data showing a positive family history of dementia in 25%-50% of FTD cases. Among these, an autosomal dominant inheritance pattern has been identified in up to 10% of patients with FTD. The pathogenesis of this disorder has been linked to multiple gene mutations, which may be present in both sporadic and familial forms. In cases where mutations are identified, over 80% of patients exhibit alterations in microtubule-associated protein tau (MAPT), progranulin (GRN), and chromosome nine open reading frame 72 (C9orf72) [6]. As a rare cause of FTD, loss-of-function mutations in TBK1 have been reported in 1.1% of cases [7]. Recent literature indicates a higher prevalence of this mutation in familial forms among European and Asian patients [8]. Molecular diagnosis in these situations is crucial, as recent studies have linked this gene alteration to rapid disease progression and overlap with MND, specifically amyotrophic lateral sclerosis (ALS). Similarly, genetic diagnosis enables timely familial counseling [7].

In this report, we present the case of a Mexican patient with a positive family history of cognitive disorders who exhibited behavioral, cognitive, and motor disturbances and was found to have a rare cause of FTD: a TBK1 mutation. This represents the first reported case at our institution, which ranks as one of Mexico's most prominent tertiary referral hospitals.

## Case presentation

We present the case of a 67-year-old Mexican male with a family history of neurodegenerative disorders. Both his paternal and maternal grandfathers, of Italian and Spanish descent, passed away at the age of 76 due to unspecified causes. During their later years, they exhibited behavioral changes and social decline without receiving a definitive diagnosis. The patient's mother died at the age of 70 due to unspecified complications related to AD. Among his five siblings, his 78-year-old sister was diagnosed with AD at the age of 73. Another sister, aged 77, showed frontotemporal atrophy starting at 75. The patient's third sister, who passed away at 75 due to pneumonia, experienced an unspecified neurodegenerative condition that began at the age of 74. His two younger sisters remain in good health. He has four sons, aged 38, 36, 31, and 29, all reported as healthy. His past medical history is unremarkable.

At the age of 57, his condition began with a gradual onset of behavioral symptoms characterized by changes in conduct, including aggression, social isolation, and apathy. These changes disrupted his family dynamics, eventually leading to a divorce. At 59, motor symptoms emerged when he experienced sudden-onset dizziness and vertigo. He was initially evaluated by an otolaryngology service and was diagnosed with benign paroxysmal positional vertigo. His medical records indicate that two years later, he developed what appeared to be a resting tremor in his left upper extremity, along with postural instability and hyperactivity. Multiple neurological services evaluated him in their quest to establish a diagnosis. These evaluations involved a range of tests, including a viral panel, liver function tests, a metabolic profile, magnetic resonance imaging (MRI), and an electroencephalogram, all of which yielded normal results. In the absence of a definitive diagnosis, symptomatic treatment for his tremor was initiated using propranolol, although no improvement was observed.

At the age of 63, new behavioral features characterized by anxiety and impulsivity emerged. In the same year, cognitive symptoms suddenly manifested, revealing disruptions in memory and temporal orientation. As a result, he received a diagnosis of parkinsonism and began treatment with levodopa/benserazide, escitalopram, and rivastigmine patches. Medical records indicated that positron emission tomography (PET) was conducted, revealing no remarkable findings. However, by the age of 64, he was transferred to a tertiary-level hospital where levodopa/benserazide was discontinued as he did not respond to dopaminergic treatment.

Over the next two years, the patient's motor disturbances continued to progress, leading to the development of oropharyngeal dysphagia. This was accompanied by a worsening of behavioral symptoms, which included abnormal eating patterns, disorganization, and increased apathy. By the age of 66, the patient had also developed flaccid dysarthria and urinary incontinence. At that point, his family chose to transfer him to a private hospital in the United States, initiating a new diagnostic approach. According to his medical record, a range of tests, including PET, electromyography, MRI, cerebrospinal fluid analysis, and microbiological cultures, were performed. The results of these tests are unknown; however, due to the absence of rigidity and bradykinesia, coupled with the presence of a left upper extremity tremor and spasticity, he was discharged with a diagnosis of Mills' syndrome. He received treatment with esomeprazole, propranolol, and quetiapine. During that year, the patient rapidly deteriorated as postural instability, dysphagia, and dysarthria progressed. At this stage, he required full assistance for activities of daily living, such as bathing and dressing. At the age of 67, he returned to Mexico and sought care at a private hospital, where the treatment he received is unknown. In the absence of improvement, he was subsequently referred to our institution.

Upon admission, we performed a complete neurological examination. On initial inspection, the patient was perceived as inattentive, unaware, and thoughtless. His Glasgow Coma Scale score was 14 points (motor: 6, verbal: 4, eye opening: 4), as he was disoriented in time and space. We then performed the Montreal Cognitive Assessment (MoCA), obtaining a score of 11 points, highlighting impairments in memory and orientation. Cranial nerve examination was remarkable for vertical gaze palsy. Facial symmetry was preserved; however, he showed decreased facial expressions. The evaluation of the rest of the cranial nerves was unremarkable. Cerebellar examination revealed postural instability and truncal ataxia. His strength and reflexes were preserved. However, muscle rigidity, bradykinesia, and a low-amplitude postural tremor in his left upper extremity were present.

He was then referred to our hospital's neuropsychologist, who performed the Neuropsi Test, a neuropsychological screening instrument designed to assess cognitive processes in both psychiatric and neurological patients, which has been developed and standardized in Mexico. His neuropsychological profile revealed probable major cognitive impairment with frontal and subcortical involvement. This was evidenced by deficits in attention and concentration, as well as impairments in memory, language, and execution. We then performed laboratory tests that included a complete blood count, coagulation tests, liver function tests, blood chemistry tests, iron kinetics, thyroid tests, vitamin B9 and B12 levels, as well as an infectious disease panel that included testing for hepatitis B virus, hepatitis C virus, syphilis, and HIV. Laboratory results showed no remarkable findings.

**Figure 1:**
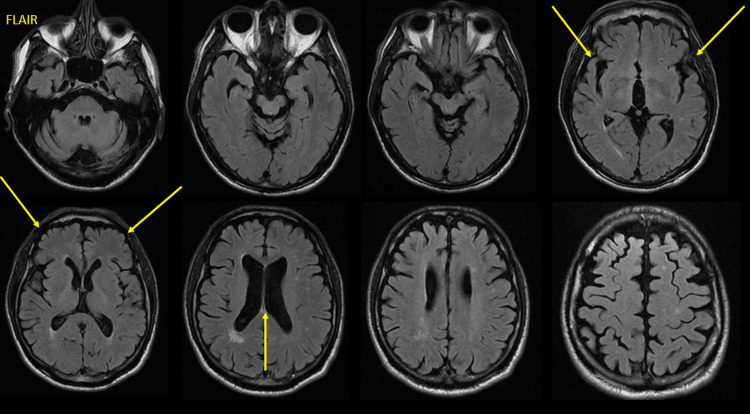
Brain MRI in FLAIR sequence, where cortical and subcortical atrophy is shown, predominantly in the frontal and temporal lobes, along with compensatory ventriculomegaly. FLAIR: fluid-attenuated inversion recovery

**Figure 2:**
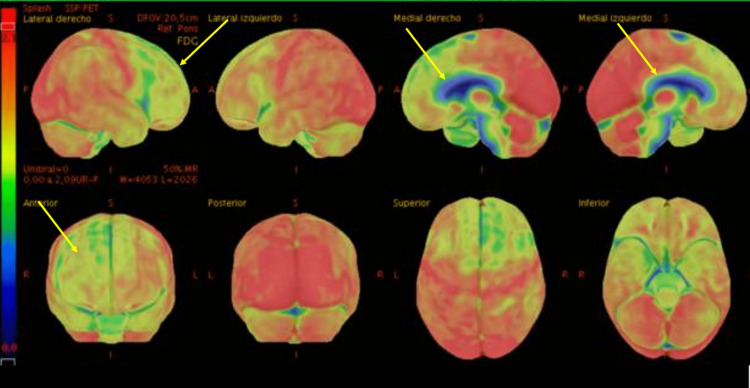
Brain 18F-FDG PET scan that shows moderate to severe bilateral prefrontal cortex hypometabolism that extends to the orbitofrontal cortex and the insular region. 18F-FDG PET: 18F-fluorodeoxyglucose positron emission tomography

**Figure 3:**
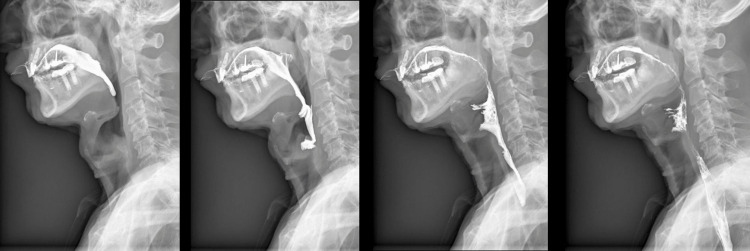
A barium swallow test shows proper coordination of pharyngeal constrictor muscles with adequate contrast propulsion.

As major cognitive impairment was established, our workup proceeded with neuroimaging studies. The patient underwent a new brain MRI (Figure 1) that revealed cerebral volume loss, predominantly in the frontal and parietal lobes, as well as compensatory ventriculomegaly. An 18F-fluorodeoxyglucose positron emission tomography (18F-FDG PET) was also performed (Figure 2), which demonstrated severe hypometabolism in the prefrontal cortex bilaterally that extended to the orbitofrontal cortex and the insular region. Severe bilateral hypometabolism of the anterior cingulate gyrus was also shown. Due to the presence of dysphagia, a barium swallow test was conducted (Figure 3). The study showed a normal oral phase with proper coordination of pharyngeal constrictor muscles and adequate contrast propulsion. Our neurophysiology department performed neuroconduction and electromyographic tests without identifying any abnormalities.

After completing the evaluation, we concluded that the patient's main diagnosis was a severe, amnestic, and multidomain cognitive impairment. As a result of a thorough examination, this diagnosis was backed up by evidenced semantic memory disturbances in screening and neuropsychological tests, along with deficits in language, visuospatial functions, and behavioral alterations spanning multiple domains. Furthermore, we established the diagnosis of a frontal lobe syndrome, which is backed up by behavioral changes shown during the patient's clinical course in conjunction with abnormal findings in imaging studies. Additionally, extrapyramidal symptoms were documented, as rigidity, bradykinesia, and tremor were present.

Due to the patient's positive family history of neurodegenerative disorders and in consideration of his clinical condition and progression, a referral was made to the genetics department for molecular assessment. We opted to request the Invitae FTD panel, a genetic test designed to identify the most common gene mutations associated with FTD. While all of the main causative FTD genes were screened, no significant mutations were identified, except for a loss-of-function mutation in TBK1. This finding established the molecular diagnosis of FTD.

After obtaining genetic results, the patient was scheduled for a follow-up consultation. Unfortunately, he did not attend subsequent appointments and was lost to follow-up. This prevented any potential adjustments in management, leaving the outcome of the case unknown.

## Discussion

FTD and its variants can overlap with atypical parkinsonian disorders, including corticobasal syndrome (CBS), progressive supranuclear palsy (PSP), and ALS [9]. This aligns significantly with the presented patient's evolution, as the onset of behavioral symptoms followed by progressive motor disturbances that highlighted postural instability and tremor early on led to a misdiagnosis of parkinsonism. Additionally, the patient also displayed cognitive symptoms, all within a short time span. However, at no point during our neurological assessment did the patient show tremors at rest. Nonetheless, a low-frequency postural tremor in his left upper extremity during shoulder flexion and elbow extension was evident during examination. This finding may explain why he did not respond to propranolol.

Causal mutations leading to FTD are most commonly found in the MAPT, GRN, and C9ORF72 genes. However, a wide range of genes may contribute as infrequent causes of FTD. Despite recent progress, the underlying genetic cause of a notable portion of familial and the majority of sporadic FTD cases remains unidentified [10]. Recently, mutations in the TBK1 gene have been associated with the development of central nervous system diseases. TBK1 is a widely expressed serine-threonine kinase that participates in diverse cellular pathways that lead to proinflammatory cytokine production, autophagic clearance of protein aggregates or pathogens, as well as basic cellular functions like growth and proliferation [11].

Loss-of-function mutations in this gene have been identified as rare causes of FTD and ALS. Mutations in TBK1 account for 0.2%-1.3% of FTD cases, 0.4%-3.4% of ALS cases, and 3.3%-4.5% of FTD-ALS cases [12]. FTD and ALS have been regarded as components of a complex neurodegenerative syndrome, with patients presenting along a wide clinical spectrum. Those who present with pure FTD display primary cognitive impairment, frequently marked by early behavioral challenges and speech disturbances. Conversely, patients with pure ALS experience motor neuron degeneration, with a deterioration in voluntary movements. Both syndromes can occur within the same family or even within the same patient [13]. Based on a recent review, more than 50% of patients who have TBK1 loss-of-function mutations have been clinically diagnosed with pure MND, primarily ALS. A smaller number of patients received diagnoses of PSP, monoparesis, and isolated lower MND. In comparison, about 25% of individuals with TBK1 mutations have been diagnosed with pure FTD. A minority of cases were classified as unspecified dementia, and roughly 20% were diagnosed with both MND-FTD and unspecified dementia [14]. 

Considering that the patient initially presented with symptoms that suggested a diagnosis of bvFTD and subsequently exhibited motor disturbances of such severity that even prompted a misdiagnosis of MND during the later stages of his disease, a mutation in the TBK1 gene corresponds well with his clinical course and aligns with findings described in recent literature. In this case, genetic testing led to a definitive diagnosis of FTD, as MND was ruled out due to the patient's normal neuroconduction and electromyographic results. The primary limitation in this report is that the patient did not attend subsequent appointments, thus preventing us from fully excluding the possibility of disease progression to MND.

## Conclusions

This case demonstrates the potential overlap between FTD and MND as a result of a TBK1 mutation. Our patient initially presented with symptoms consistent with bvFTD, followed by the onset of motor symptoms and cognitive impairments. His symptom progression ultimately led to a series of misdiagnoses and inappropriate treatments. This situation highlights the challenges associated with achieving an accurate diagnosis in FTD cases, given that patients may display a broad range of signs and symptoms that initially appear nonspecific but ultimately develop into a defined disease. It is crucial to emphasize that during the initial stages of these cases, imaging studies may appear normal and neurological examinations might yield unremarkable results, much like what occurred with this patient.

In our clinical practice, we emphasize the significance of conducting a comprehensive neurological examination and a thorough review of the patient's medical history, including familial and hereditary backgrounds. This approach is essential for patients presenting with suggestive cognitive decline symptoms, regardless of their age, as it can offer valuable insights into possible genetic causes of their disorder.
